# Differential *HFE* Gene Expression Is Regulated by Alternative Splicing in Human Tissues

**DOI:** 10.1371/journal.pone.0017542

**Published:** 2011-03-03

**Authors:** Rute Martins, Bruno Silva, Daniela Proença, Paula Faustino

**Affiliations:** Departamento de Genética, Instituto Nacional de Saúde Dr. Ricardo Jorge, Lisboa, Portugal; Centre de Regulació Genòmica, Spain

## Abstract

**Background:**

The pathophysiology of *HFE*-derived Hereditary Hemochromatosis and the function of HFE protein in iron homeostasis remain uncertain. Also, the role of alternative splicing in *HFE* gene expression regulation and the possible function of the corresponding protein isoforms are still unknown. The aim of this study was to gain insights into the physiological significance of these alternative HFE variants.

**Methodology/Principal Findings:**

Alternatively spliced *HFE* transcripts in diverse human tissues were identified by RT-PCR, cloning and sequencing. Total *HFE* transcripts, as well as two alternative splicing transcripts were quantified using a real-time PCR methodology. Intracellular localization, trafficking and protein association of GFP-tagged HFE protein variants were analysed in transiently transfected HepG2 cells by immunoprecipitation and immunofluorescence assays.

Alternatively spliced *HFE* transcripts present both level- and tissue-specificity. Concerning the exon 2 skipping and intron 4 inclusion transcripts, the liver presents the lowest relative level, while duodenum presents one of the highest amounts. The protein resulting from exon 2 skipping transcript is unable to associate with β2M and TfR1 and reveals an ER retention. Conversely, the intron 4 inclusion transcript gives rise to a truncated, soluble protein (sHFE) that is mostly secreted by cells to the medium in association with β2M.

**Conclusions/Significance:**

*HFE* gene post-transcriptional regulation is clearly affected by a tissue-dependent alternative splicing mechanism. Among the corresponding proteins, a sHFE isoform stands out, which upon being secreted into the bloodstream, may act in remote tissues. It could be either an agonist or antagonist of the full length HFE, through hepcidin expression regulation in the liver or by controlling dietary iron absorption in the duodenum.

## Introduction

Maintaining iron homeostasis is essential, as both iron deficiency and iron excess are associated with cellular and organismal dysfunction. Iron homeostasis is dependent of a tight link between body iron requirements, storage, recycling from macrophages, and intestinal iron absorption. However, how this complex mechanism is controlled remains largely to be understood.

**Figure 1:**
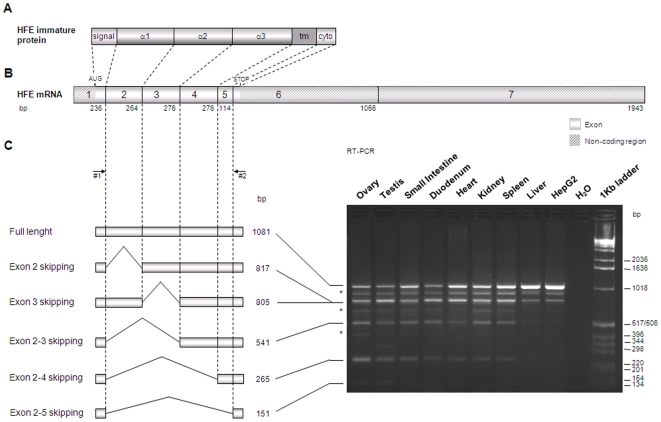
Splicing forms of *HFE* gene in several human tissues. (**A**) Schematic representation of the HFE immature protein. The distinct patterns depict HFE protein domains: the signal peptide (signal), the three extracellular domains (α1, α2 and α3), the transmembrane domain (tm) and the cytoplasmic tail (cyto). (**B**) Schematic representation of the major *HFE* transcript. The length of exons is presented in base pairs (bp). (**C**) A RT-PCR using total RNA from eight tissues and HepG2 cell line was performed using primers #1 and #2 (their relative position is indicated with arrows) and results are shown on the right. The products obtained for each tissue were cloned into the pCR®-TOPO-XL® vector and sequenced. On the left are schematic representations of the alternative splicing forms identified as well as their length in bp. The asterisks (*) identify bands corresponding to PCR artefacts as a result from DNA hybrid chains.

HFE is a major histocompatibility complex (MHC) class I-like protein that is mutated in Hereditary Hemochromatosis (HH; OMIM 235200), a common autosomal recessive disorder of iron metabolism [1]. The disease is characterized by excessive intestinal iron absorption and iron deposition in organs such as liver, heart and pancreas, potentially leading to cirrhosis, hepatocellular carcinoma, diabetes, cardiac failure and arthritis [2]. HFE is a transmembrane protein formed by six distinct domains: a signal peptide, three extracellular domains (α1, α2 and α3), a transmembrane region and a short cytoplasmic tail [1] (Figure 1A). It assembles with its chaperone beta2-microglobulin (β2M) to form an heterodimer expressed at the cell surface. The most common HH-associated *HFE* mutation, C282Y, abrogates the disulfide bond in the protein α3 domain and prevents its binding to β2M and cell surface presentation [3].

HFE protein has been detected in various cell types. It is expressed throughout the gastrointestinal tract as well as in macrophages and monocytes [4], [5]. In the human liver, HFE was shown to be present on Kupffer cells and endothelium [6]. In a variety of transfected cells, HFE co-localizes with transferrin receptor 1 (TfR1) at the cell surface and in perinuclear compartments, namely the endosomal compartment [7]–[9]. At cell surface, both HFE and diferric-iron-loaded transferrin (Fe2-Tf) recognize overlapping regions on TfR1, which results in competition for binding to this receptor [10]. However, HFE is also able to interact with the liver-specific TfR1 homologue, TfR2 [11]. Recently, it was proposed that, under normal conditions, HFE is partitioned between TfR1 and TfR2, and an increase in Fe2-Tf saturation results in stabilization of TfR2 protein and degradation of *TfR1* mRNA [12]. Under these conditions, HFE should shift away from TfR1 towards TfR2, so TfR2-HFE complex is possibly part of the iron sensing complex involved in the induction of the iron regulatory hormone hepcidin [13], [14].

The *HFE* gene (formerly known as *HLA-H*) is located at 6p21.3 and its genomic structure resembles other MHC class I molecules [1]. It is known that alternative splicing mechanism is a widespread mean for producing polypeptide diversity from a single gene [15], . Accordingly, alternative splicing is a common process of producing MHC class I protein isoforms. For instance, *HLA-G*, which is a non-typical MHC class I protein that presents significant structural homology to *HFE*, shows alternative splicing expression regulation and some of the isoforms produced have specific biological functions [17], [18]. Previous studies have shown that *HFE* gene expression is subjected to alternative splicing as well [19]–[22]. The predominant *HFE* transcript has about 4.2 kb, but additional transcripts have also been reported, which seem to differ in both level- and the tissue- or cellular-specificity. However, the identification of *HFE* alternative transcripts, their tissue-specificity and abundance, as well as the biological significance of the corresponding isoforms, remains to be clarified.

As a consequence of alternative splicing, soluble protein isoforms can be originated, assuming in some cases, an important regulatory role in physiological processes. Actually, a splice variant of *HFE* mRNA was described [19] and, although not studied at protein level, it was suggested that the corresponding soluble peptide might regulate cellular iron transport. In addition, an artificial β2M-HFE monochain, trying to mimic a soluble HFE (sHFE), was constructed and it was observed that it effectively reduces Tf uptake into cells [23]. However, this did not correlate to any changes in TfR1 or ferritin synthesis, in contrast to the normal HFE-induction. These findings of an incongruent soluble β2M -HFE monochain activity suggest that sHFE may act differently. Nevertheless, the existence and the biological function of a putative sHFE isoform remained elusive.

In this study, we have characterized several *HFE* splicing alternative transcripts in a variety of human tissues, their relative abundance and tissue-specificity. Raising the hypothesis that some of the corresponding protein variants might have a biological role, we analysed those resulting from exon 2 skipping and intron 4 inclusion. By studying their intracellular localization, trafficking and assembly, we gained insights about their physiological significance. Therefore, we have demonstrated that a sHFE isoform is secreted into the medium, maintaining its association to the chaperone β2M.

## Results

### *HFE* mRNA is alternatively spliced in different human tissues

Pools of total RNA from eight human tissues (heart, duodenum, small intestine, liver, spleen, kidney, ovary and testis) and from HepG2 cell line were retrotranscribed to cDNA. A PCR approach using a specific primer pair (Table 1, primers #1 and #2) in which primer #2 includes the native HFE' stop codon in the 5′ part of HFE exon 6, allowed the amplification of the previously predicted coding region of *HFE* gene (full length) and theoretically of all the alternative transcripts resulting from partial or total exon skipping or intron inclusion that present the native stop. At least eight bands could be observed in some lanes of the representative gel photograph (Figure 1B and 1C). To identify the corresponding *HFE* transcripts, the RT-PCR products from all samples were cloned and sequenced. As expected, we found the correctly spliced full length transcript represented by the 1081 base pair (bp) fragment. Additionally, we identified two transcripts resulting from the skipping of a single exon: one corresponds to the *HFE* exon 2 skipping (817 bp fragment) and the other to the exon 3 skipping (805 bp fragment). Three other transcripts were also found as a result of multiple exon skipping: exon 2–3 (541 bp fragment), exon 2–4 (265 bp fragment) and exon 2–5 (151 bp fragment). Moreover, three other bands (indicated with an asterisk) could be seen in most of the lanes of the gel (Figure 1C). However, they were proved, by direct sequencing, to be artefact fragments of DNA hybrid chains formed during PCR assays.

**Table 1:** DNA oligonucleotides used in the current work.

Primer	Localization	Sequence (5′→3′)
#1	Exon 1	ATGGGCCCGCGAGCCAGGCCG
#2	Exon 6	GTCTCCTTCCCACAGTGAGTCTGCAGGCTG
#3	Exon 4	GAACATCACCATGAAGTGGCTGAAGG
#4	Exon 5	GAACAAAATTCCAATGAACAAGATGACG
#5	Exon 6	CTACGTCTTAGCTGAACGTGAGTGA
#6	Exon 6	TGTCTCCTTCCCACAGTGAGTCT
#7	Exon 1/3	TGCAGGGTGTGGGACTGCAGCAAGCG
#8	Intron 4	GGCAATCAAAGGCTTTAACTTGCTTTT
#9	Intron 4/Exon 5	CCAGACGGTGAGGGCTCTAA
#10	Exon 1	TTTTGGTACCATGGGCCCGCGAGCC
#11	Exon 6	TTTTGGATCCCACTCACGTTCAGCTAAGACGTAGTGCCC
#12	Exon 4	GGGAAGAGCAGAGATATACGTACCAGGTGGAGCACCC
#13	Exon 4	GGGTGCTCCACCTGGTACGTATATCTCTGCTCTTCCC
#14	Intron 4	TTTTGGATCCCACATACCCCAGATCACAATGAGG

The full length as well as most of the alternatively spliced *HFE* transcripts were found in all the analysed tissues (Figure 1C). As exon 2 and exon 3 skipping transcripts were not distinguished in the gel due to their similar molecular weight, their presences in all tissues were confirmed by additional RT-PCRs (Figure S1, Table S1). Only the exon 2–5 skipping seems to be tissue-specific, since it is only present in the gonads, small intestine, duodenum and heart (Figure 1C). Most of the alternative transcripts were also found in HepG2 cells, with the exception of the exon 2–4 and exon 2–5 skipping transcripts.

In all of these alternative transcripts, exons are totally skipped without generating frameshifts. However, in some of them, a single amino acid change occurs in the new exon-exon junction. As an example, the alternative transcript with a complete deletion of exon 2 results in a 260-aa protein variant where the arginine 26 changes to glutamine.

**Figure 2:**
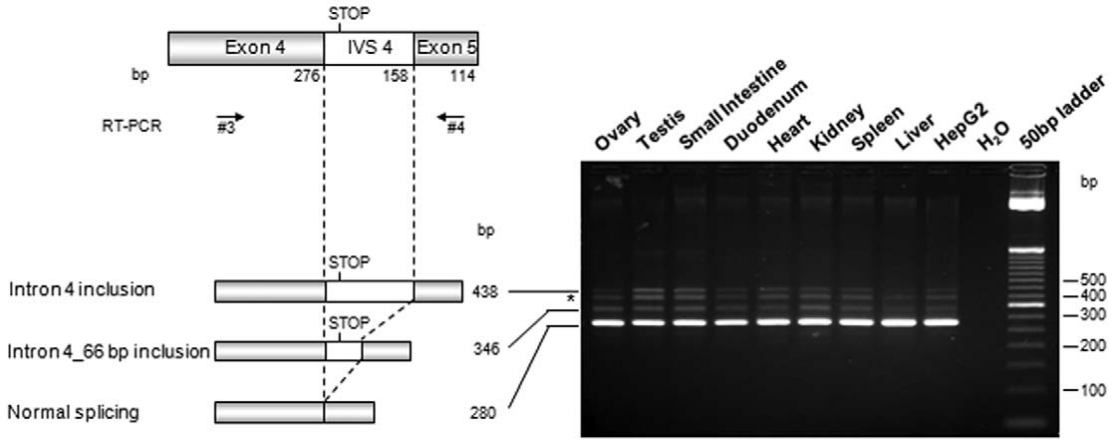
Expression of the intron 4 inclusion *HFE* splice transcript in several human tissues. A specific RT-PCR to amplify the region between *HFE* exon 4 to 5 using total RNA from eight tissues and HepG2 cells was performed. These amplified products were cloned into the pCR®-TOPO-XL® vector for automated sequencing and further identification. A schematic representation of the *HFE* gene exon 4 to 5 is presented on the left. The intron 4 (IVS4) contains a stop codon (TGA) six nucleotides from the exon 4 boundary. The position of the primers (#3 and #4) used in the PCR and schematic representations of the identified alternative splicing forms are revealed. Correspondence between these splicing forms and the PCR amplification products is shown, along with their length in bp. The asterisks (*) identify bands corresponding to PCR artefacts as a result from DNA hybrid chains.

In order to improve the screening for alternatively spliced *HFE* transcripts, we performed a search for one previously described, resulting from the inclusion of intron 4 [19]. So, a specific RT-PCR using primers located at exons 4 and 5 was performed in all tissues and HepG2 cells (Figure 2). The amplified products were cloned and sequenced. Besides the normally spliced, two additional *HFE* transcripts were identified, one resulting from the total intron 4 inclusion (438 bp fragment) and other, not previously published, resulting from the first 66 bp intron 4 inclusion (346 bp fragment) (Figure 2). These alternative transcripts were observed in all analysed tissues as well in HepG2 cells. On the other hand, an additional band was presented in the gel (indicated with an asterisk) that was once again proved by direct sequencing to be a PCR artefact. The same RT-PCR approach was replicated using minus RT controls for all the tissues. Result allows to discard any DNA contamination (Figure S2).

A RT-PCR encompassing exon 1- intron 4 was also performed in all tissues to confirm the correct splicing of their upstream coding region (data not shown). So, potentially both transcripts encode the same and putatively soluble HFE peptide since it would not have the transmembrane domain (encoded by exon 5) and the cytoplasmatic tail (encoded by exon 6). Therefore, after α3 domain it would have two extra C-terminal amino acids, glycine and methionine, encoded by the 5′ sequence of intron 4.

### Absolute and relative quantification of *HFE* exon 2 skipping and intron 4 inclusion transcripts reveal a tissue-specific pattern

In order to further characterize two of the identified alternative spliced transcripts, resulting from exon 2 skipping or intron 4 insertion, absolute quantification of the total *HFE* mRNA along with these mentioned transcripts was performed by quantitative real-time PCR (qRT-PCR). These two species were chosen because of the clear opposite outcome that, hypothetically, they could have. The exon 2 skipping corresponding protein will miss the α1 domain, which is essential for the interaction with TfR1. In contrast, the intron 4 insertion corresponding protein will have the three α domains required for interaction with TfR1 and β2M but will not contain the transmembrane region and the cytoplasmic tail.

The approach used was an accurate two-step real-time RT-PCR described for appropriate measurement of several low-abundance mRNA splice variants [24]. Four independent reverse transcription assays were performed, using total RNA from each tissue, which allowed to conclude about inter-assay variation. Since *HFE* exon 6 is present in the full length as well as in all the abnormally spliced transcripts, primers located in this exon were used to quantify the total *HFE* transcripts. For the same reason, in order to corroborate this quantification data, a similar procedure was made using primers located in exon 1 (data not shown). Specific primer sets were designed based on differences in the splicing pattern of each alternative transcript, where the sequence is unique. For instance, to quantify the exon 2 skipping transcript, a specific primer was designed spanning the exon 1/3 boundary, whereas to quantify the intron 4 inclusion a primer spanning intron 4/exon 5 boundary was used. An absolute quantification method was performed using serial dilutions of plasmid constructs as standards (8×10^5^ – 80 copies), previously obtained by cloning the RT-PCR fragments into the pCR®-TOPO-XL vector. Each reaction was done in triplicate. Linear regression analysis of each standard curve from all plates was used to quantify transcript levels. The correlation coefficients ranged from 0.986 to 0.999, indicating low intra-assay variation. Quantification of total and alternative transcripts was also done in triplicate for each cDNA, and all standard deviations were less than 0.38 *C*_t_.

**Figure 3:**
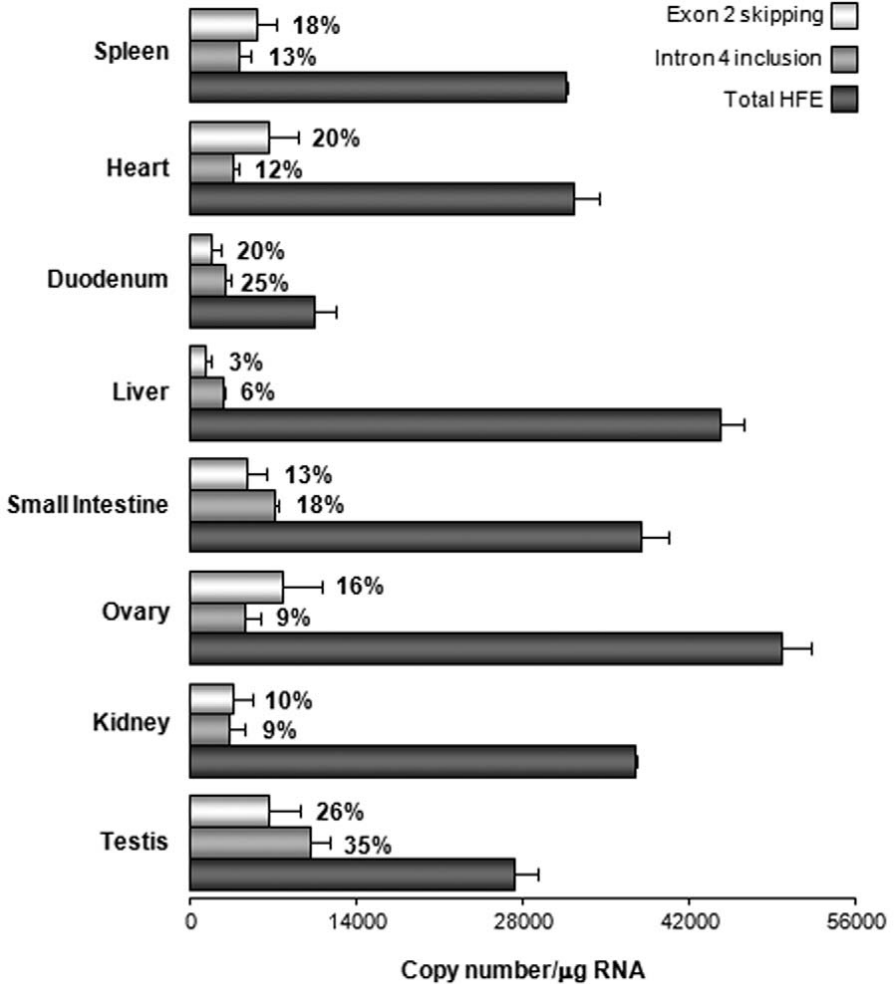
Absolute and relative quantification of the exon 2 skipping and intron 4 inclusion *HFE* splice transcripts. Absolute quantification of total *HFE* and of two alternative splicing transcripts was performed by the absolute quantification method using serial dilutions of plasmids as standards. The histogram shows the mean and standard deviations from four independent experiments. The relative levels of the alternatively spliced transcripts resulting from exon 2 skipping and intron 4 inclusion are shown as a percentage of the total *HFE* (defined as 100%).

The qRT-PCR methodology performed allowed the quantification of total *HFE* as well as the two alternative *HFE* transcripts in all analysed tissues (Figure 3). The standard deviations presented in these experiments reflect similar RT efficiency, which indicates low inter-assay variation. Absolute quantification (presented as *HFE* copy number/µg RNA) showed that ovary and liver have the highest level of total *HFE* mRNA while, on the contrary, the smallest amount is present in duodenum (Figure 3). Comparing the two tissues thought to be targets for HFE function, liver presents an amount of total *HFE* mRNA approximately 4.3-fold higher than duodenum.

Similarly, the relative quantification of the exon 2 skipping and intron 4 inclusion transcripts also revealed a differential expression in the analysed tissues. For instance, the liver presents the lowest relative amount of both transcripts (3 and 6%, respectively) and, on the contrary, testis (26 and 35%, respectively) and the duodenum (20 and 25%, respectively) are the tissues where they are more expressed (Figure 3).

### HFE variants present distinct subcellular localization

In an attempt to characterize the cellular localization of the corresponding HFE protein splice variants, *HFE* cDNAs (corresponding to the full length *HFE* mRNA, exon 2 skipping and intron 4 inclusion transcripts) were tagged to the GFP gene in the pEGFP-N1 vector (Clontech). In addition, a construct containing the full length *HFE* C282Y mutant was made to be used as a dysfunctional control.

**Figure 4:**
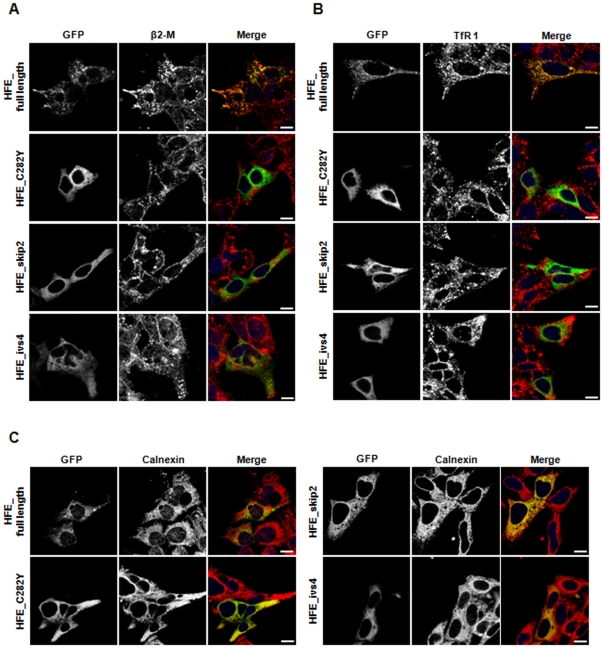
Cellular localization of HFE splice variants by immunofluorescence analysis. HepG2 cells were transfected with 2 µg of pEGFP_HFE_full length, pEGFP_HFE_C282Y, pEGFP_HFE_skip2 and pEGFP_HFE_ivs4 constructs. Twenty-four hours later, cells were submitted to an immunofluorescence assay. HFE protein variants distribution was compared with the location for (**A**) beta2-microglobulin (rabbit anti-β2M polyclonal antibody), (**B**) transferrin receptor 1 (mouse anti-human TfR1 monoclonal antibody) and (**C**) endoplasmic reticulum (rabbit anti-calnexin polyclonal antibody). Images were acquired using a 488-nm laser for GFP (green) and a 532-nm laser for the previously described antibodies (red). Nuclei were stained with DAPI (blue). Bars represent 10 µm.

Since we previously have shown that HepG2 cells endogenously express the *HFE* exon 2 skipping and intron 4 inclusion transcripts, they provide a suitable model to further characterize the corresponding transgenic proteins. This artificial system was developed due to the lack of suitable anti-HFE antibodies available for endogenous protein detection. Therefore, these cells were transiently transfected with the mentioned constructs. Confocal microscopy analysis of the subcellular localization of (i) the full length HFE protein, (ii) proteins related to the two splice transcripts, and (iii) the mutated HFE_C282Y was performed using antibodies against β2M, calnexin (an endoplasmic reticulum, ER, marker) and TfR1 (Figure 4). Nuclei were stained with DAPI. Under these conditions, HFE_full length protein presents mostly a perinuclear and cell membrane distribution. As expected, it co-localizes with β2M and TfR1 proteins. On the contrary, also as expected, HFE_C282Y is not present at the cell surface and has a diffuse cytoplasmic localization. It seems not to co-localize with β2M and TfR1, being retained in the ER, as revealed by the calnexin co-localization (Figure 4). As well, the intracellular distribution of HFE_skip2 variant is similar to the one obtained for the HFE_C282Y variant, since it appears to be co-localized with calnexin but not with β2M and TfR1. Concerning the HFE_ivs4 variant, it presents a scattered intracellular distribution and is apparently absent from the cell membrane. It seems not to co-localize with either β2M or TfR1 and to be present in the ER (Figure 4). Since the fluorescence microscopy data only hints about the localization of these variants, immunoprecipitation assays were performed to all the HFE variants to further clarify these results.

### Immunoprecipitation assays reveal a soluble and secreted HFE protein isoform

**Figure 5:**
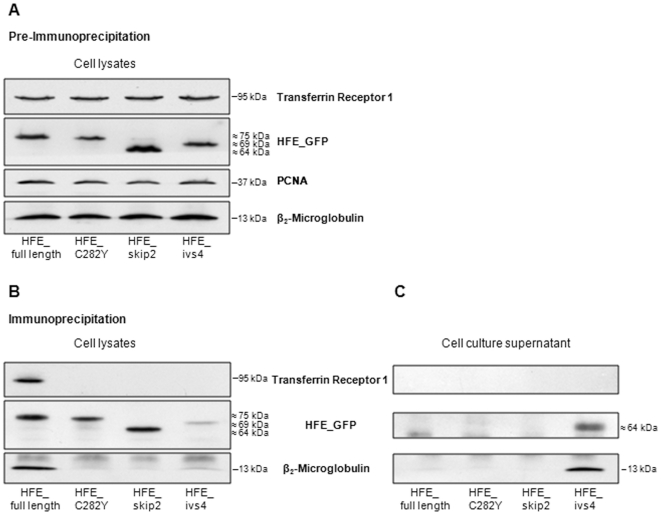
Immunoprecipitation assays of transfected HFE splice variants. HepG2 cells were transfected with pEGFP_HFE_full length, pEGFP_HFE_C282Y, pEGFP_HFE_skip2 or pEGFP_HFE_ivs4 constructs. (**A**) Protein expression in HepG2 transfectants prior to immunoprecipitation. (**B**) Cell lysates and (**C**) cell media were subjected to immunoprecipitation using a mouse anti-GFP monoclonal antibody and G-agarose beads. Blots were incubated with anti-TfR1, -GFP and -β2M antibodies for protein detection. The predicted molecular mass of the proteins is indicated in kDa.

**Figure 6:**
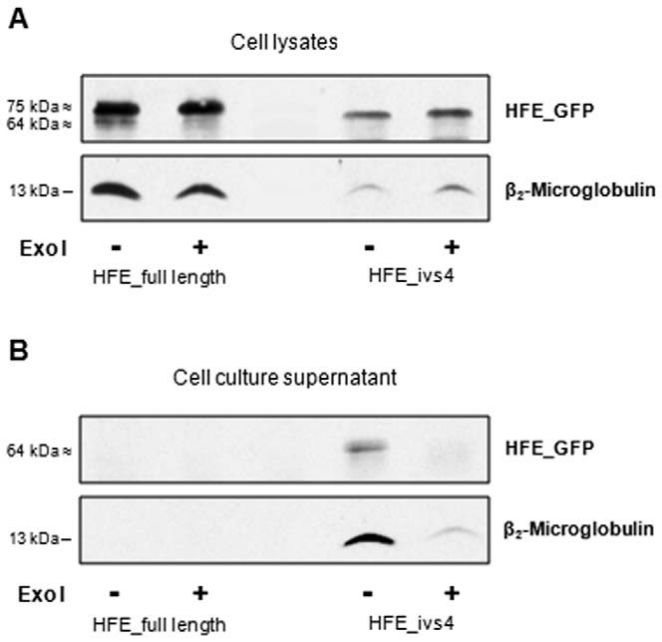
sHFE cell secretion inhibition. HepG2 cells were transfected with pEGFP_HFE_full length or pEGFP_HFE_ivs4 constructs along with Exo I (500 nM) incubation. (**A**) Cell lysates and (**B**) cell media were subjected to immunoprecipitation using a mouse anti-GFP monoclonal antibody and G-agarose beads. Blots were incubated with anti-GFP and -β2M antibodies for protein detection. The predicted molecular mass of the proteins is indicated in kDa.

To confirm our protein co-localization data observed in immunofluorescence assays, proteins obtained from cell lysates as well as from cell culture supernatants were subjected to immunoprecipitation assays using a mouse anti-GFP antibody (Figure 5). In cell lysate experiments, the HFE_full length protein is bound to β2M and TfR1, while HFE_C282Y does not co-immunoprecipitate with these proteins. Similarly, HFE_skip2 variant does not bind to either β2M or TfR1. In addition, it can be observed that HFE_ivs4 variant seems to be present at low level in cell lysates (of different type of cells) in association with β2M but not with TfR1 (Figure 5A and B) These results are in agreement with those obtained by immunofluorescence experiments (Figure 4). The same procedures carried out in the corresponding cell culture supernatants reveal that HFE_full length, HFE_C282Y and HFE_skip2 are absent from the culture media (Figure 5C). Conversely and interestingly, the HFE_ivs4 variant is clearly shown in the culture supernatant in association with its chaperone β2M. In order to ascertain that the presence of the sHFE-GFP in cell supernatant is due to cell secretion and it is not a basic result of cell leakage, a secretion inhibitor was used (Exo 1). The results presented in Figure 6 shows that when we inhibited secretion we almost not detect sHFE-GFP in the extracellular supernatant, even though the protein is detected intracellularly. Furthermore, the secretion of sHFE-GFP was also confirmed to occur in other types of transfected cell lines: HuH7, HEK293, HeLa and CaCo2 (the latter results are shown in Figure S3). In all cases, this isoform is largely secreted to cell medium remaining linked to the β2M, as shown by immunoprecipitation experiments. This result reveals for the first time a soluble form of HFE-GFP protein (sHFE- GFP) which is secreted to the cell medium, as it lacked the transmembrane and cytoplasmic domains.

## Discussion

Alternative mRNA splicing is a complex post-transcriptional mechanism that enables the generation of multiple mRNA products from a single gene, increasing transcriptome and proteome complexity. So, by this way, a single gene can produce proteins with different properties and functions, which might differ in a tissue- or developmental stage-specific manner. Here we report that, in addition to full length *HFE*, at least seven alternatively spliced *HFE* transcripts are expressed in several human tissues, differing in the level- and the tissue-specificity. Some studies had already shown that *HFE* gene is subjected to alternative splicing processes [19]–[22]. However, the precise characterization of the alternative transcripts, their tissue-specificity and abundance, as well as the intracellular localization and biological significance of the corresponding protein isoforms remain largely to be clarified.

The search and identification of *HFE* transcripts in a range of human tissues (heart, duodenum, small intestine, liver, spleen, kidney, ovary and testis) allowed us to distinguish, besides the full length, seven alternative splicing transcripts. Among them, the one resulting from the first 66 bp intron 4 inclusion was not previously published. Some other *HFE* transcripts have been previously described but were not found in this analysis, e.g. an in-frame deletion of the first 69 bp of exon 2 was identified in HepG2 cells by Sanchez et al. [22]. Nevertheless, this transcript was also not found in studies performed by other authors in several cell lines and in peripheral blood lymphocytes [20], [21].

In this work, two transcripts were chosen to be further characterized as they have the potential to produce opposite structural protein outcomes, those resulting from the exon 2 skipping and intron 4 inclusion. So, an absolute and a relative quantification of total *HFE* transcripts as well as of the two mentioned alternatively spliced transcripts were performed using samples of RNAs' pools from the diverse tissues. We took advantage of a qRT-PCR strategy, as it is the most sensitive method to ascertain gene expression levels. It offers a substantially higher sensitivity than other conventional methodologies previously used for *HFE* transcripts quantification, as southern blot of RT-PCR products [19], RT-PCR [20] and northern blot [21], [22]. As we used pools of RNAs from several healthy individuals, results represent an average of *HFE* expression in each tissue, and do not reflect inter- or intra- individual variability. Using this approach, we observed that total *HFE* mRNA expression varies among tissues. Apart from the gonads, we found that the liver has the highest total *HFE* expression. On the contrary, duodenum shows the lowest *HFE* expression of the tissues tested. Curiously, the liver, besides presenting a high level of total *HFE* mRNA, has the lowest amount of the studied *HFE* alternative transcripts. We can hypothesize that in the liver, wild type (full length) HFE is playing an important role in iron metabolism. On the contrary, duodenum, the tissue where the total *HFE* expression is the lowest, presents a relative high level of the studied alternative transcripts (approximately 45% of the total). One may speculate that these alternative transcripts may have a significant function in this tissue. Having in mind the two models of HFE possible action, the crypt model [25] and the hepcidin regulation model [13], [14], [26], we attempted to further understand the different tissue-level of *HFE* transcripts by studying the cellular localization of the overexpressed corresponding proteins.

Concerning the *HFE* exon 2 skipping transcript, which was already described in hepatic, colon and ovary cell lines [20], [22], here we demonstrate its presence at different levels in several human tissues. Regarding its corresponding protein, the extracellular α1 domain encoded by exon 2 is lacking, and therefore it is unable to bind to TfR1 [27]. Our immunofluorescence and immunoprecipitation results, besides confirming that HFE_skip2 is not associated with TfR1, also revealed no interaction with the β2M chaperone and consequent ER retention. It is possible to conclude that this variant apparently does not have any cellular function, being probably degraded by the cell proteolytic system.

The other alternative transcript selected to be further characterized is the one resulting from the intron 4 inclusion. It presents a tissue-specific regulation, since a relatively more abundant level of it is found in the testis and in the duodenum than in other tissues. On the other hand, the lowest level is found in the liver. Accordingly, a previous study also found different levels of this transcript in duodenal and liver biopsies of control and iron overload patients [19].

Analysing the sequence of the intron 4 inclusion transcript we can observe the presence of a stop codon (TGA) in the open reading frame, six nucleotides from the exon 4 boundary. Therefore, this alternative transcript would be expected to be degraded by the nonsense-mediated mRNA decay mechanism (NMD), which is a post-transcriptional surveillance mechanism responsible for the rapid degradation of transcripts harbouring a premature stop codon. In this way, NMD limits the production of C-terminally truncated polypeptides and protects the cell from their possible deleterious dominant-negative or gain-of-function effects [28]. Nevertheless, this alternative *HFE* transcript is easily detected by PCR approaches, thus it may escape NMD or be only partially degraded. A parallel situation occurs concerning another MHC class I gene, the *HLA-G* gene, where a similar alternative transcript is generated (also including a premature stop codon in a similar position) giving rise to a soluble protein with a recognized biological function [18]. In fact, it is known that as a consequence of the alternative splicing mechanism, soluble protein isoforms can be originated, assuming in some cases, an important regulatory role in physiological processes. Actually, some years ago, an alternatively spliced *HFE* transcript due to the intron 4 inclusion was described [19]. It was detected at a relatively high level in duodenum biopsies of normal individuals or with secondary iron overload. On the contrary, absence or low presence was observed in duodenal biopsies of HH-C282Y patients. Although not studied at protein level, it was suggested that the corresponding soluble peptide might regulate cellular iron transport. Here, we positively show for the first time, a soluble HFE protein isoform lacking the transmembrane domain and the cytoplasmic tail, due to the in frame premature stop codon present at intron 4 (Figure 2). Since it has an intact α3 domain (encoded by exon 4), it can bind its chaperone β2M, be correctly folded, conducted to the cell surface and secreted to the cell medium (Figure 5 and Figure 6). Furthermore, the sHFE isoform secretion was verified to occur (in addition to HepG2 cell line) in other diverse types of transfected cell lines (HuH7, HeLa, HEK293 and CaCo-2), which suggests that it is a widespread event.

Altogether, our results show that *HFE* gene post-transcriptional regulation is clearly affected by a tissue-dependent alternative splicing mechanism. By the approach performed, the alternative transcripts analysed revealed a distinct level of expression amongst tissues. However, it would be important to assess their interindividual variability for each tissue. Consequently, it would be possible to establish the actual degree of variation between tissues or organs and thus hypothesize about the physiological importance of the *HFE* alternative transcripts.

Regarding the results obtained for the sHFE protein variant, although its function still requires further investigation, we propose that this variant may play a role in systemic iron metabolism regulation. For instance, the sHFE produced in several tissues may be secreted into the bloodstream and thus bind to cell surface expressed transferrin receptors (TfR1 or TfR2). Therefore, it could act as an agonist or an antagonist of the wild type HFE on hepcidin expression activation in the liver, by controlling the dietary iron absorption in the duodenum (modulating the expression of iron-related transporters) or by exerting a role on iron recycling by macrophages. The function of the sHFE secreted by duodenum may not be crucial for the systemic iron homeostasis under iron steady state conditions, as already shown for the full length HFE [29]. However, it cannot be disregarded a putative sHFE response to iron imbalance conditions. Therefore, it will be important to investigate if sHFE serum levels vary with changes in body iron stores, such as in iron overload disorders (such as HH) or in iron deficiency conditions. If so, we can also hypothesise that a sHFE isoform might be developed as an useful therapeutic agent in the treatment of iron-related disorders.

## Materials and Methods

### First strand cDNA synthesis and Polymerase Chain Reaction

First strand cDNA synthesis was performed using 3 µg of total RNA from each RNA pool of eight human tissues (BD Clontech or Ambion) and HepG2 cell line (DSMZ ACC 180, Germany), an equivalent mixture of random primers and oligo(dT)_12–18_ and the SuperScript® II Reverse Transcriptase (RT; Invitrogen), according to the manufacturer's instructions. Using RNAs from different vendors did not introduce variability in quantification experiments, since ovary RNAs from Clontech and Ambion were simultaneously tested and similar results were obtained. A polymerase chain reaction (PCR) covering the entire *HFE* coding region was performed to the synthesized cDNAs using primers #1 and #2 (Table 1). A specific PCR to amplify the region between *HFE*'s exon 4 to 5 was done using primers #3 and #4.

All products obtained in both PCRs were cloned into the pCR®-TOPO-XL® vector (Invitrogen), sequenced with BigDye terminator v1.1 sequencing standard kit using the ABI Prism 3100 automatic sequencer (Applied Biosystems).

### Quantitative real-time PCR

The quantification of the alternatively spliced *HFE* transcripts was conducted using real-time PCR performed on an ABI Prism 7000 Sequence Detection System. Primers were designed to amplify specific amplicons for the total *HFE* (exon 6 – primers #5 and #6), for the exon 2 skipping (exon 1–3 boundary, primers #1 and #7) and for the inclusion of intron 4 (intron 4 - exon 5 boundary, primers #8 and #9) using the ABI Primer Express software (Applied Biosystems).

Synthesis of cDNA from each tissue was carried out as before. Each cDNA sample was diluted 5-fold and 5 µL added to 5 µmol primers and SYBR Green Master Mix (Applied Biosystems). The cycling parameters were: 10 min at 95°C, followed by 40 cycles of 15 sec at 95°C and 1 min at 65°C. Quantification of gene expression was performed by the absolute standard curve method.

### Plasmid constructs

The cloning of *HFE* cDNA into the pEGFP-N1 (Clontech) took advantage of the previously obtained RT-PCR products cloned into pCR®-TOPO-XL vector. A construct already containing the total *HFE* cDNA was used to amplify the entire *HFE* coding sequence with primers #10 and #11, containing the *Kpn*I and *Bam*HI linkers next to the translation start and stop codons (that was modified in order to allow fusion to GFP open reading frame), respectively. Both pEGFP-N1 vector and PCR product were digested with the *Kpn*I and *Bam*HI endonucleases to clone the full length *HFE* cDNA fused to GFP (pEGFP_HFE_full length). The same method was performed to create pEGFP_HFE_skip2 construct, to mimic the exon 2 skipping transcript. To clone the C282Y mutant control, the pEGFP_HFE_full length was amplified using mutagenic primers #12 and #13, along with QuikChangeTM Site-Directed Mutagenesis Kit (Stratagene). In order to clone the splice variant in which the intron 4 is included, an antisense primer containing a *Bam*HI linker (primer #14) along with primer #10 were used to amplify this exon 1 - intron 4 fragment using cDNA from small intestine as a template for the PCR. Once more, the *Kpn*I and *Bam*HI endonucleases were used to create the pEGFP_HFE_ivs4 construct. Final sequence analysis was performed to confirm that all constructs contained the correct sequence.

### Cell culture and transient transfections

HepG2 cells (a human cell line with hepatocyte characteristics), commercially obtained (DSMZ, ACC 180, Germany) were maintained in RPMI 1640 medium supplemented with 10% (v/v) fetal bovine serum in a 37°C/5% CO_2_ atmosphere. For transient transfections, cells were seeded in 35 mm plates at a confluence of 5×10^5^ or 8×10^5^ cells per well, for immunofluorescence or immunoprecipitation assays, respectively. Twenty-four hours after seeding, 2 µg of the pEGFP_HFE constructs were used together with Lipofectamine 2000 Transfection Reagent (Invitrogen) or Lipofectamine™ LTX and PLUS™ Reagents (Invitrogen), for immunofluorescence and immunoprecipitation assays, respectively. Transfections were performed following the manufacturer's instructions. When required, cells were incubated with Exo I (500 nM) immediately after transfection. For immunoprecipitation and western blot analyses, cells and supernatant were harvested 48 hours post-transfection, whereas for immunofluorescence assays, cells were analyzed 24 hours after transfection.

### Immunofluorescence assays

Twenty-four hours after transfection, cells were washed in PBS, fixed in methanol at −20°C and then washed again. Subsequently, cells were permeabilized and blocked simultaneously in a PBS solution containing FBS 10% and Triton X-100 0.5% for 30 min at room temperature. Incubation with the primary antibodies [rabbit anti- β2M (Abcam) at 1∶200 dilution; mouse anti-TfR1 (Zymed) at 1∶100 dilution or rabbit anti-calnexin (SantaCruz Biotechnology) at 1∶50 dilution] was performed for one hour at room temperature. Afterwards, another incubation was made using Cy3-conjugated secondary antibodies, anti-mouse or anti-rabbit (Jackson Immunoresearch Laboratories), both at 1∶100 dilution. Nuclei were stained with 10 µg/mL DAPI (Sigma). Coverslips were then mounted in VectaShield (Vector Laboratories) and sealed. Images were acquired with the 405-nm, 488-nm and 532-nm laser lines using a Leica DMI 4000B confocal microscope and processed with Leica Analysis Software.

### Immunoprecipitation assays

Forty-eight hours after transfection, both cell media (≈2 mL) and platted cells were harvested. Firstly, cell media were centrifuged 5 min at 2000 rpm. To their supernatant and cells, 150 µL of lysis buffer [50 mM Tris-HCl at pH 7.5, 1% (v/v) NP-40, 100 mM NaCl, 10% (v/v) glycerol, 10 mM MgCl_2_, and a protease inhibitor cocktail (Sigma)] was added on ice. The media and cell lysates were cleared by centrifugation and an aliquot of 20 µL (Pre-IP) transferred to 2× Laemmli buffer. To the remaining supernatant, 5 µL of mouse GFP monoclonal antibody (Roche) was added. After one hour of incubation at 4°C, 60 µL of G-agarose beads slurry (1∶1 in lysis buffer) was added and incubated overnight. Beads were spun down and a 20 µL aliquot of the supernatant (Post-IP) was added to 2× Laemmli buffer. Beads were washed and protein resuspended in 2× Laemmli buffer. These lysates, together with pre- and post-IP aliquots were analyzed by western blot.

### Western blot analysis

Proteins from cell lysates or from cell culture supernatants were resolved in a 12% SDS-PAGE and transferred to PVDF membranes (Bio-Rad), which were blocked using a 15% (m/v) TBST-Milk solution. Membranes were probed using mouse anti-GFP (Abcam) at 1∶10000 dilution, rabbit anti- β2M (Abcam) at 1∶500 dilution or mouse anti-TfR1 (Zymed) at 1∶500 dilution. For pre-IP lysates, a mouse anti-PCNA antibody (Calbiochem) was used as a loading control. Detection was carried out using secondary peroxidase-conjugated anti-mouse IgG (Bio-Rad) at 1∶4000 dilution or anti-rabbit IgG (Bio-Rad) at 1∶3000 dilution antibodies, followed by chemiluminescence assays.

## Supporting Information

Figure S1 **Expression of** ***HFE*** **exon 2 and exon 3 skipping splice transcripts in several human tissues and HepG2 cell line.** (**A**) A specific RT-PCR to amplify the region between *HFE* exons 1 to 3 using total RNA from eight tissues and HepG2 cell line was performed to evaluate the presence of exon 2 skipping. A schematic representation of the *HFE* gene exons 1 to 3 is presented on the left. The position of the primers (#S1 and #S2) used in the PCR and schematic representations of the identified alternative splicing forms are revealed. (**B**) A specific RT-PCR to amplify the region between *HFE* exons 2 to 4 using total RNA from eight tissues and HepG2 cell line was performed to evaluate the presence of exon 3 skipping. A schematic representation of the *HFE* gene exons 2 to 4 is presented on the left. The position of the primers (#S3 and #S4) used in the PCR and schematic representations of the identified alternative splicing forms are revealed. Correspondence between these splicing forms and the PCR amplification products is shown, along with their length in bp. The asterisks (*) identify bands corresponding to PCR artefacts as a result from DNA hybrid chains.
(TIF)

Figure S2 **Control PCR for genomic DNA contamination of RNA samples.** A PCR to amplify the region between *HFE* exon 4 to 5 using cDNA from liver and HepG2 (first two lanes) or total RNA from eight tissues was performed to evaluate the possible genomic DNA contamination of the RNA samples. Splicing forms and the corresponding PCR amplification products are shown.
(TIF)

Figure S3 **Immunoprecipitation assays of transfected HFE splice variants.** CaCo-2 (above) and HeLa (below) cells were transfected with pEGFP_HFE_full length, pEGFP_HFE_C282Y, pEGFP_HFE_skip2 or pEGFP_HFE_ivs4 constructs. Cell lysates and cell media were subjected to immunoprecipitation using a mouse anti-GFP monoclonal antibody and G-agarose beads. Blots were incubated with anti-GFP and -β2M antibodies for protein detection. The predicted molecular mass of the proteins is indicated in kDa.
(TIF)

Table S1 Supplementary DNA oligonucleotides used in the current work.
(DOCX)
