# Supplementary figures and images for: Differential HFE Gene Expression Is Regulated by Alternative Splicing in Human Tissues
Source: PLoS One. 2011 Mar 3;6(3):e17542. doi: 10.1371/journal.pone.0017542 (PMC3048171; doi:10.1371/journal.pone.0017542)

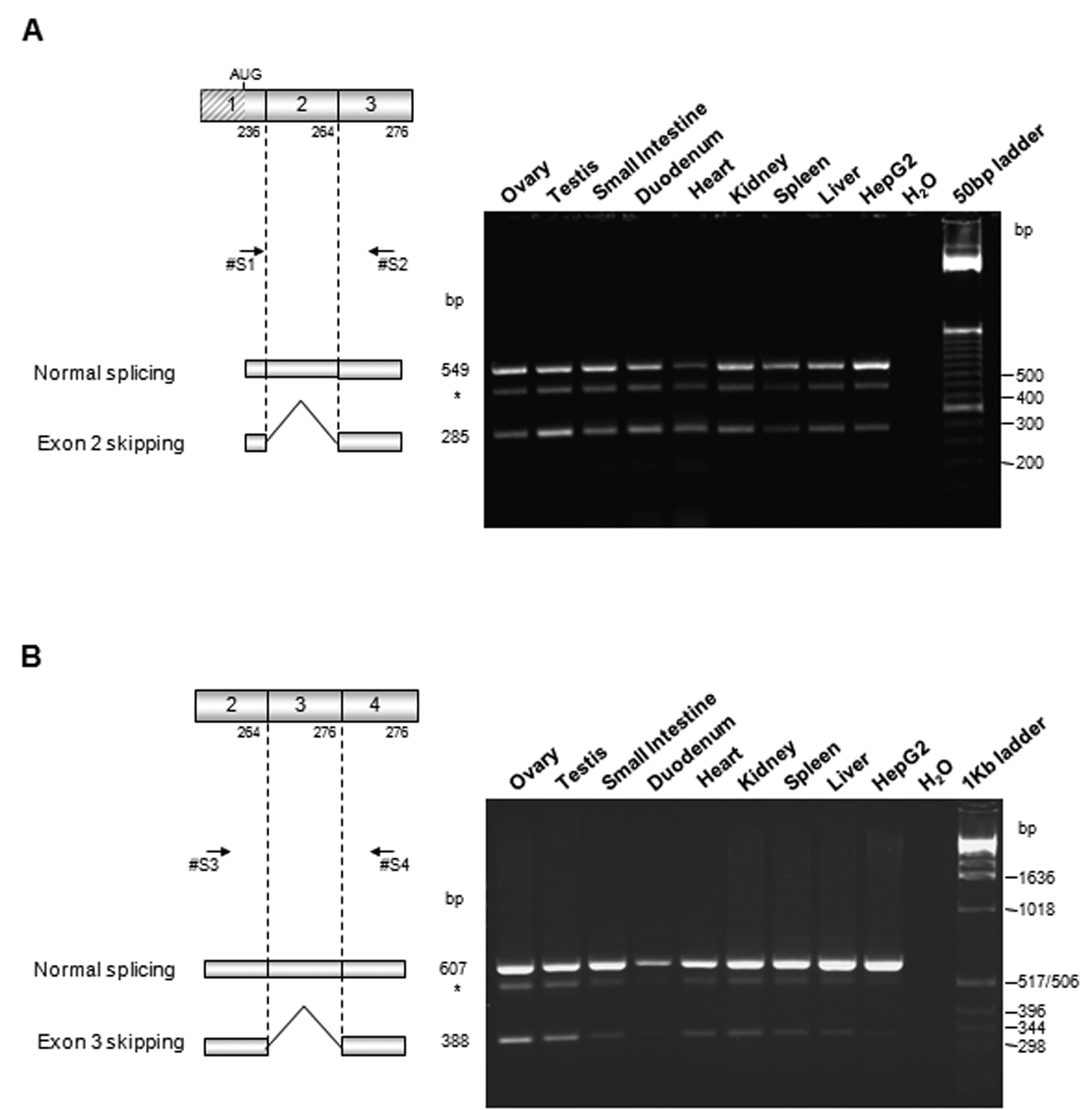

Supplement: Figure S1 — Expression of HFE exon 2 and exon 3 skipping splice transcripts in several human tissues and HepG2 cell line. (A) A specific RT-PCR to amplify the region between HFE exons 1 to 3 using total RNA from eight tissues and HepG2 cell line was performed to evaluate the presence of exon 2 skipping. A schematic representation of the HFE gene exons 1 to 3 is presented on the left. The position of the primers (#S1 and #S2) used in the PCR and schematic representations of the identified alternative splicing forms are revealed. (B) A specific RT-PCR to amplify the region between HFE exons 2 to 4 using total RNA from eight tissues and HepG2 cell line was performed to evaluate the presence of exon 3 skipping. A schematic representation of the HFE gene exons 2 to 4 is presented on the left. The position of the primers (#S3 and #S4) used in the PCR and schematic representations of the identified alternative splicing forms are revealed. Correspondence between these splicing forms and the PCR amplification products is shown, along with their length in bp. The asterisks (*) identify bands corresponding to PCR artefacts as a result from DNA hybrid chains. (TIF) [file pone.0017542.s001.tif]

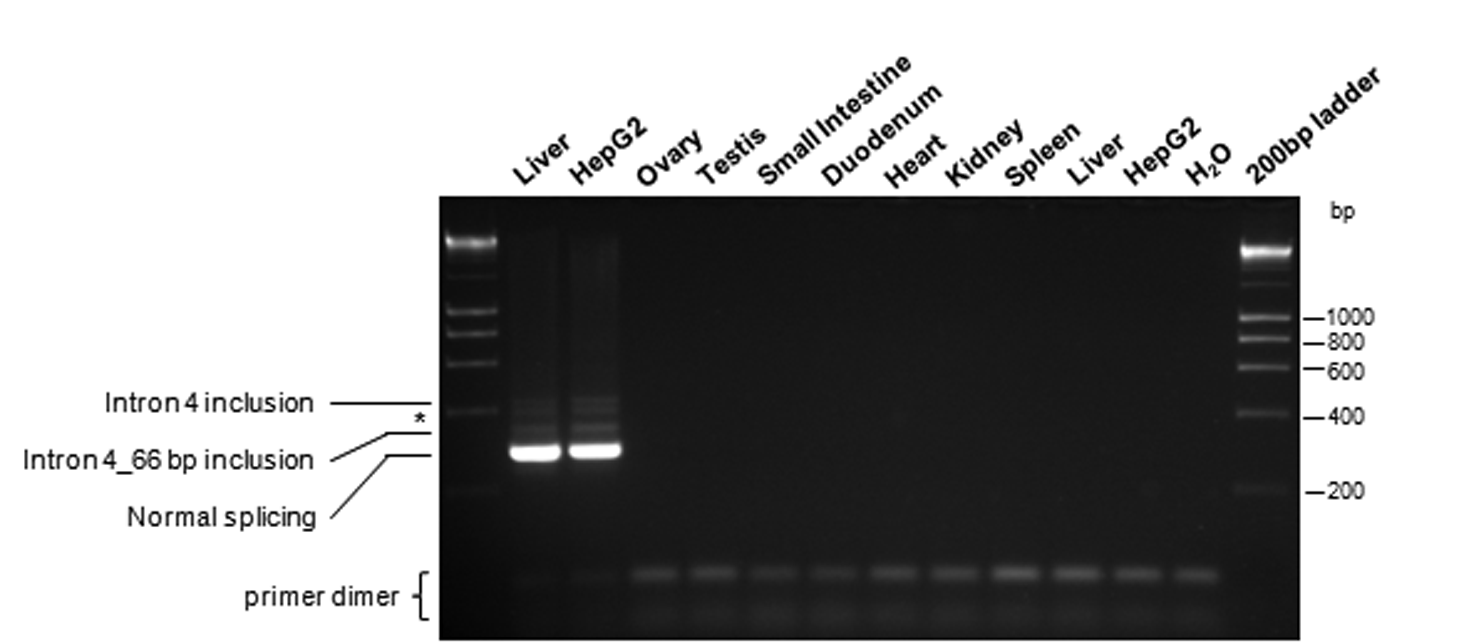

Supplement: Figure S2 — Control PCR for genomic DNA contamination of RNA samples. A PCR to amplify the region between HFE exon 4 to 5 using cDNA from liver and HepG2 (first two lanes) or total RNA from eight tissues was performed to evaluate the possible genomic DNA contamination of the RNA samples. Splicing forms and the corresponding PCR amplification products are shown. (TIF) [file pone.0017542.s002.tif]

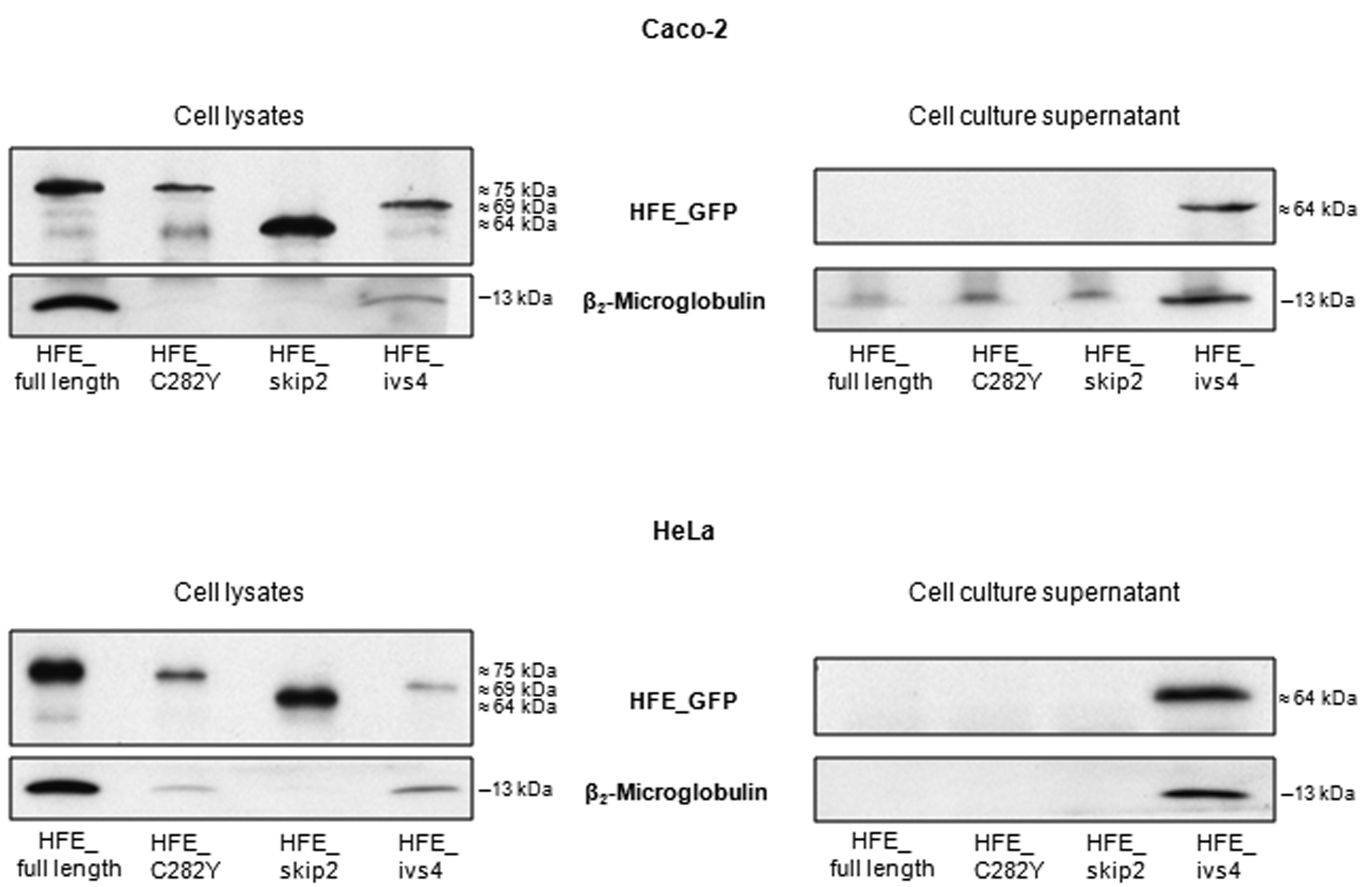

Supplement: Figure S3 — Immunoprecipitation assays of transfected HFE splice variants. CaCo-2 (above) and HeLa (below) cells were transfected with pEGFP_HFE_full length, pEGFP_HFE_C282Y, pEGFP_HFE_skip2 or pEGFP_HFE_ivs4 constructs. Cell lysates and cell media were subjected to immunoprecipitation using a mouse anti-GFP monoclonal antibody and G-agarose beads. Blots were incubated with anti-GFP and -β2M antibodies for protein detection. The predicted molecular mass of the proteins is indicated in kDa. (TIF) [file pone.0017542.s003.tif]
