# Supplementary material for: Differential HFE Gene Expression Is Regulated by Alternative Splicing in Human Tissues
Source: PLoS One. 2011 Mar 3;6(3):e17542. doi: 10.1371/journal.pone.0017542 (PMC3048171; doi:10.1371/journal.pone.0017542)
Supplement: Table S1 — Supplementary DNA oligonucleotides used in the current work. (DOCX) [file pone.0017542.s004.docx]

**SUPPLEMENTARY INFORMATION**

**Table S1 - Supplementary DNA oligonucleotides used in the current work**

| Primer | Location | Sequence (5’ 🡪 3’) |
| --- | --- | --- |
| #S1 | Exon 1 | ATGGGCCCGCGAGCCAGGCCG |
| #S2 | Exon 3 | CTGTTCTGCCTGGCCCGAATCTTGTGCCTTTCC |
| #S3 | Exon 2 | GCTGTTCGTGTTCTATGATCATGAGAGTCGCCGTGTGG |
| #S4 | Exon 4 | GGGCAATACGTCTTTAGGTTCGAACTCCTTGGCATCC |
